# Comparison of Tumor- and Bone Marrow-Derived Mesenchymal Stromal/Stem Cells from Patients with High-Grade Osteosarcoma

**DOI:** 10.3390/ijms19030707

**Published:** 2018-03-01

**Authors:** Louis-Romée Le Nail, Meadhbh Brennan, Philippe Rosset, Frédéric Deschaseaux, Philippe Piloquet, Olivier Pichon, Cédric Le Caignec, Vincent Crenn, Pierre Layrolle, Olivier Hérault, Gonzague De Pinieux, Valérie Trichet

**Affiliations:** 1 Laboratoire d’étude des sarcomes osseux et remodelage des tissus calcifiés, INSERM UMR 1238, Université de Nantes, PhyOS, 44034 Nantes CEDEX 1, France; lrlenail@hotmail.com (L.-R.L.N.); meadhbhbrennan@gmail.com (M.B.); philippe.rosset@univ-tours.fr (P.R.); cedric.lecaignec@chu-nantes.fr (C.L.C.); vincrenn@gmail.com (V.C.); pierre.layrolle@inserm.fr (P.L.); gonzague.dubouexic@univ-tours.fr (G.D.P.); 2 Centre Hospitalier Régional Universitaire de Tours, Service de Chirurgie Orthopédique 2, Faculté de Médecine de Tours, Université de Tours, 37044 CEDEX 9 Tours, France; 3 Harvard School of Engineering and Applied Sciences, Harvard University, Cambridge, MA 02138, USA; 4 STROMA Lab, INSERM U1031, Etablissement Français du Sang Occitanie, Université de Toulouse, 31432 Toulouse, France; frederic.deschaseaux@efs.sante.fr; 5 Centre Hospitalier Universitaire de Nantes, Service de Génétique Médicale, Faculté de Médecine de Nantes, 44034 CEDEX 1 Nantes, France; philippepil@gmail.com (P.P.); Olivier.PICHON@chu-nantes.fr (O.P.); 6 Centre Hospitalier Universitaire de Nantes, Service de Chirurgie Orthopédique, Faculté de Médecine de Nantes, Université de Nantes, 44034 CEDEX 1 Nantes, France; 7 Centre Hospitalier Régional Universitaire de Tours, Service d’Hématologie Biologique, 37044 CEDEX 9 Tours, France; olivier.herault@univ-tours.fr; 8 National Center for Scientific Research (CNRS) GDR 3697, 75020 Paris, France; 9 National Center for Scientific Research (CNRS) ERL 7001 LNOx, 37032 CEDEX 1 Tours, Université de Tours, 37044 Tours, France; 10 Centre Hospitalier Régional Universitaire de Tours, Hôpital Trousseau, Service d’Anatomie Pathologique, Faculté de Médecine de Tours, Université de Tours, 37044 CEDEX 9 Tours, France

**Keywords:** osteosarcoma, mesenchymal stem cells, cancer stem cells, spheres

## Abstract

Osteosarcoma (OS) is suspected to originate from dysfunctional mesenchymal stromal/stem cells (MSC). We sought to identify OS-derived cells (OSDC) with potential cancer stem cell (CSC) properties by comparing OSDC to MSC derived from bone marrow of patients. This study included in vitro characterization with sphere forming assays, differentiation assays, cytogenetic analysis, and in vivo investigations of their tumorigenicity and tumor supportive capacities. Primary cell lines were isolated from nine high-grade OS samples. All primary cell lines demonstrated stromal cell characteristics. Compared to MSC, OSDC presented a higher ability to form sphere clones, indicating a potential CSC phenotype, and were more efficient at differentiation towards osteoblasts. None of the OSDC displayed the complex chromosome rearrangements typical of high grade OS and none of them induced tumors in immunodeficient mice. However, two OSDC demonstrated focused genomic abnormalities. Three out of seven, and six out of seven OSDC showed a supportive role on local tumor development, and on metastatic progression to the lungs, respectively, when co-injected with OS cells in nude mice. The observation of OS-associated stromal cells with rare genetic abnormalities and with the capacity to sustain tumor progression may have implications for future tumor treatments.

## 1. Introduction

Osteosarcoma (OS) is the most prevalent primitive bone cancer [1], bone multiple myeloma excluded, and occurs mostly in children and young adults [2]. The long bone metaphysal zone is the most prevalent OS site, with 50% of cases occurring around the knee area; consequently, surgical tumor resection is often followed by reconstructive surgery for bone and joint replacement. In high-grade OS patients, metastases are mostly pulmonary and radiographically detectable in 15% to 20% of cases at diagnosis; they are associated with poor prognosis (30% 5-year disease free survival rate) [3,4]. Before the introduction of neo adjuvant chemotherapy, surgery alone achieved less than 12% 5-years disease free survival rate and usually consisted of amputation. With the advent of neoadjuvant poly-chemotherapy, the cure rate was improved to 70% for patients with non metastatic OS at diagnosis [5,6,7,8]. However, no significant progress has been made since [8] and OS remains a major cause of death by cancer in adolescent and young adult patients [9].

OS is defined by the World Health Organization’s histologic classification of bone tumors as a “Malignant neoplasm in which the neoplastic cells produce bone” [2]. In 1967, Dahlin established a classification for conventional OS based on their predominant cancer cell constituents: osteoblastics, chondroblastics, and fibroblastics [10]. In addition to this tumor tissue heterogeneity, high-grade OS is characterized by complex genetic rearrangements [11,12,13] with no specific marker, contrarily to Ewing’s Sarcoma [14], and to low grade OS (<6% of all OS) which demonstrate a simple genetic profile characterized by amplified sequences of the chromosome 12 region 12(q13q15), including oncogenes *MDM2* and *CDK4* [15]. Concerning high grade OS, such massive chromosome rearrangements likely result from chromothripsis [16]. This process could occur early in the tumor development and may induce cell transformation through the amplification of oncogenes, combined with a loss of tumor-suppressor genes expression. However, cells bearing such huge chromosome rearrangements are usually not capable of sustained cell division or survival. The presence of cancer stem cells (CSC) in OS has been hypothesized to explain tumor heterogeneity, its chemotherapy resistance, and its high capacity to metastasize [17]. Moreover, CSC could be the origin of early OS progenitors that could then undergo cell division and chromothripsis. There are multiple lines of evidence in favor of Mesenchymal Stromal/Stem Cell (MSC) being the cell of origin of OS [18]. In fact, the osteoblast, which is the only cell capable of producing an osteoid matrix, derives from MSC. Moreover, MSC are multipotent cells with the potential to give rise to chondrocytes and fibroblasts [17,19,20], corresponding with the variety of the different OS subtypes. Therefore, OS is likely to originate at an earlier osteoblastic MSC differentiation stage [21] and recently human MSC have been successfully transformed into OS-inducing cells following Retinoblastoma protein gene (*pRb,* anti oncogene located on 13q14.2) silencing combined with *c-Myc* (oncogene located on 8q24.2) overexpression [22]. Interestingly *SOX2* (a stemness marker and inducer) was up-regulated in those transformed MSC, similarly to in one of the rare OS-derived primary cell lines that induced tumors in mice (tumorigenicity properties) [23].

Evidence to support the CSC origins of OS was first presented by Gibbs et al. [24]. Potential OS-CSC were isolated from five biopsies of untreated OS due to their ability to form spherical clones in non-adherent and serum free culture. The cell surface markers associated with MSC were identified, including CD105 on 30–50%, and CD44 on 75–100%, of CSC. Those potential CSC also showed their abilities to differentiate into adipogenic and osteoblastic lineages. However genomic instability and properties of tumor induction were not tested. Only two primary OS-derived cell lines have demonstrated tumorigenicity properties, the BCOS and OSA-13 cell lines from Adhikari et al. and Skoda et al. respectively [23,25]. However, the karyotypes were not investigated for the OS-inducing primary cells or for the corresponding parental OS. In contrast, Brune et al. described that only mesenchymal progenitors with no chromosomal aberrations, rather than tumor cells, were obtained from five out of six fresh OS biopsies [13].

Regarding the undoubtedly key roles of CSC in chemotherapy resistance, tumor recurrence, and metastasis progression, the isolation and biological characterization of such cells in OS may be of great interest in order to understand the underlying mechanisms of the disease and aid in overcoming the present treatment failures. Since MSC are the suspected cells of OS origin, we performed a comparative study of nine high grade OS-derived cells (OSDC) with either mesenchymal stromal/stem cells (MSC) derived from the bone marrow of six out of those nine OS patients, or with healthy donors. This study included functional tests of in vitro properties, including clone formation in methylcellulose, osteoblast/adipogenic differentiation, and gene expression analysis. Additionally, all OSDC were analyzed for conventional karyotypes and specifically followed by Comparative Genomic Hybridization (CGH) arrays when required. Furthermore, OSDC were injected alone in nude and/or severe combined immunodeficiency (SCID) mice to assess tumorigenicity and co-injected with an OS cell line (MNNG-HOS) in nude mice to investigate tumor-supportive activity.

## 2. Results

### 2.1. Clinical Characteristics of Nine Patients with High-Grade OS and Sample Processing

The study cohort included 9 patients, 7 males and 2 females, diagnosed with high-grade OS, mostly conventional (predominately osteoblastic, but a few chondroblastic and fibroblastic subtypes were also detected), except for one telangiectatic case, see Table 1. The average age at diagnosis was 19 years old. The lower limb, in the knee region, was the most prevalent location of the disease (6/9). Surgical resection prevented local recurrence in all cases. A poor response rate to neo adjuvant poly-chemotherapy was observed for 5 patients. Metastatic disease was detected for 2 patients at diagnosis (20% of cases) and OS spread to the lungs for a further 3 patients without metastasis at diagnosis, but with a poor response rate to pre-operative poly-chemotherapy. Considering this clinical data, our cohort is small but representative of high-grade OS patients, both in terms of disease presentation, tumor location and characteristics, and disease evolution.

Three different methods of conservation and dissociation were used to treat tumor samples before cell seeding in standard culture conditions (Table 2). Independently of the process, primary cells were successfully derived from all OS samples. However, we observed a higher rate of cell recovery using enzymatic dissociation compared with mechanical dissociation, but no difference between the Collagenase I or MACS human tumor dissociation kit. Three tumor samples, OSDC-4, 5, 6 (corresponding to OSDC derived from patients identified as 4–6) were frozen before cell dissociation and seeding. We observed that the amplification time of primary cells was longer from frozen samples compared to fresh samples. We measured up to 80% cell death after freezing, despite employing cryoprotective medium, compared to cell viability observed for the same sample before freezing. Finally, during transfer, tumor samples were conserved in MACS Tissue Storage Solution at 4 °C for a duration of 24–72 h and a satisfactory rate of cell recovery was obtained. Immediately after cell recovery from tumor samples, cells were either seeded in adhesion culture conditions on tissue culture plastic, or in methylcellulose to induce the anchorage independent formation of sphere clones.

### 2.2. Similarities between Osteosarcoma Derived Cells (OSDC) and Mesenchymal Stromal/Stem Cells (MSC) Derived from the Bone Marrow of Either OS Patients, or Healthy Donors

**Figure 1:**
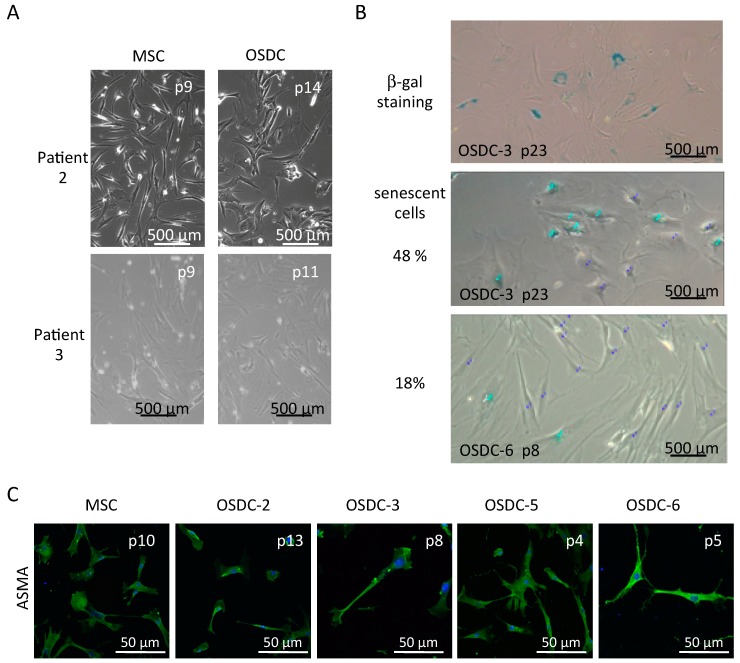
Microscopic observation of primary cell lines derived from bone marrow (MSC) or OS samples (OSDC) on treated culture dishes. Representative images are shown for MSC and OSDC at indicated passages (p). (**A**) Optical microscopy observation of adherent cells in standard culture conditions; (**B**) Optical microscopy observation of X-gal staining (upper panel, senescent cells have blue cytoplasmic color) and quantification of senescent cells (middle and bottom panels). Quantification was performed on 50 nuclei identified by two associated nucleoli using ImageJ. Non-senescent cells and senescent cells are shown with dark or light blue points, respectively; (**C**) Optical fluorescent microscopy immunodetection of alpha-smooth muscle actin (ASMA) (green), with nuclei colored in blue.

Similar to MSC derived from the bone marrow, OSDC were adherent to plastic culture flasks and presented an elongated fibroblastic form (Figure 1A). They grew quickly initially (dividing once every 34 h until passages 6 to 8) then slowly, before senescence occurred, identified by the observation of larger cells confirmed by β-galactosidase activity. Senescence was observed primarily around passages 10 to 12 for MSC, whereas β-galactosidase activity was detected from passage 8 for OSDC-6 to passage 20 for OSDC-1 (Figure 1B).

Stromal cells derived from bone marrow following adherence culture on tissue culture plastic are commonly referred to as MSC, whereas stromal cells derived from tumors may be likened to cancer-associated fibroblasts (CAF). Alpha-smooth muscle actin (ASMA) has been traditionally used to identify CAF [26]. In our study, ASMA was similarly detected by immunofluorescence on OSDC and MSC (Figure 1C). Not surprisingly, both types of primary cell lines were positive for Vimentin, confirming their mesoderm origin and they showed no differences in fluorescent intensity following immunofluorescence detection. 

**Figure 2:**
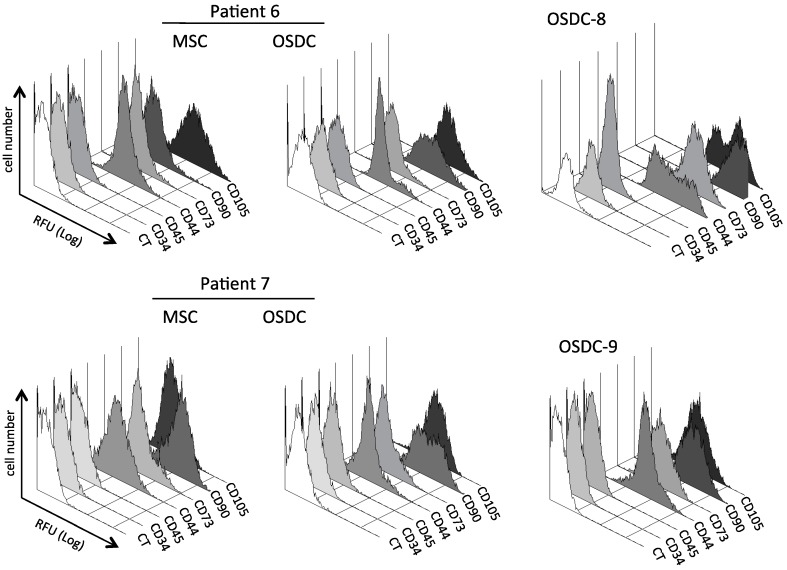
Histograms of relative fluorescent unites (RFU) for cluster of differentiation (CD) antigens on primary cell lines derived from OS samples (OSDC) or bone marrow (MSC) obtained from patients 6–9. RFU identified as CT were acquired with the control isotype (IgG-PE). MSC from patient 8 and 9 were not available. Colors are used to simplify reading.

With regard to characteristic MSC surface markers, despite variability during tumor conservation and dissociation, all primary cells selected by plastic adherence were positive for the three markers CD73, CD90, and CD105, which are commonly recommended for identification of MSC, and negative for at least two hematopoietic cell markers (CD34, CD45) (Figure 2; Table 3). MSC and OSDC lines were also positive for CD44, which is a receptor to hyaluronic acid, an extracellular matrix molecule. As shown in Table 3, ratios of the mean fluorescence intensity observed for seven differentiation clusters were in a similar range for 9 OSDC compared to 6 MSC lines derived from the iliac bone marrow of OS patients. However, as shown in Figure 2, the CD marker profiles of MSC lines at passage 3 were usually more homogenous compared to the corresponding OSDC lines. Indeed, heterogeneous cell populations were observed within OSDC-7 and OSDC-6 for CD90 detection and within OSDC-8 for CD44 and CD105 markers.

Following culture selection in methylcellulose-containing medium, spheres were dissociated and isolated cells were seeded on tissue culture treated plastic dishes for amplification. Sphere-derived cells were adherent to plastic flasks but they demonstrated a long dividing duration (once every 30 to 100 days). Consequently only 6 sphere-derived OSDC and no sphere-derived MSC lines were obtained in sufficient quantity to analyze their expression of surface markers (CD) (Table 3). We observed no major difference of CD expression, neither between 2 sphere-selected clones derived from OSDC-1-patient’s OS, nor between 3 clones derived from OSDC-2-patient’s OS (supplementary Table S1). The ratios of mean fluorescent intensity observed for CD34, CD45, CD44, CD73, CD90, and CD105 were similar for sphere-derived OSDC and MSC lines compared to adhesion-selected OSDC and MSC lines (Table 3). Overall, none of the MSC or OSDC appeared as immortalized cell lines. All primary cell lines presented stromal cell characteristics and retained a similar combination of surface markers that characterizes MSC [27] when cultured with basic fibroblast growth factor (bFGF) in adhesion or in anchorage-independent conditions.

### 2.3. Differences in Stemness Properties between OSDC and MSC

**Figure 3:**
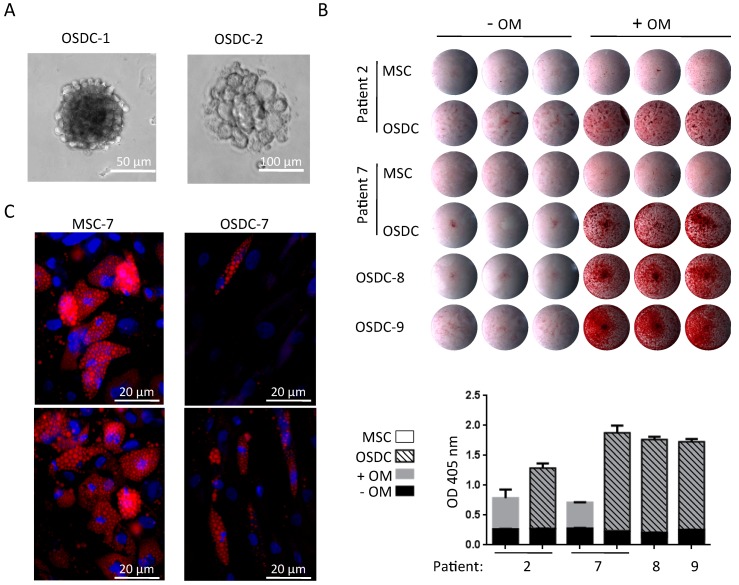
Osteosarcoma derived cells (OSDC) in anchorage-independent, osteogenic or adipogenic culture conditions. (**A**) Optical microscopy observation of sphere clones in anchorage-independent culture conditions; (**B**) Calcium deposition observed with alizarin red staining following 21 days of cell culture with (+OM) or without (-OM) osteogenic induction media. Images of stained-culture wells are shown for OSDC or MSC plated in triplicate for each culture condition. Bound alizarin red was solubilized and optical density (OD) was measured for each well. The histogram represents mean values with standard deviation of each triplicate; (**C**) Representative images of MSC and OSDC from patient 7 following 14 days of cell culture with adipogenic induction medium are shown. Adipocytes containing small Nile Red-positive lipid droplets are easily observed (red vesicles) whereas undifferentiated cells were not labeled with Nile Red. Nuclei were counterstained (blue color) with 4′,6-diamidino-2-phenylindole (DAPI).

Sphere clone development from OSDC-2 was observed only with bFGF, whereas no sphere was detected without bFGF in the semi-solid medium (Supplementary Figure S1; Figure 3A). Following these observations, bFGF was always added during the sphere assays. In these conditions, all OSDC except OSDC-3, formed sphere clones, with a 67 × 10^−7^ to 65 × 10^−5^ ranging rate (Table 2). Three patient-corresponding MSC did not form sphere clones. All OSDC, excluding OSDC-3, formed sphere clones with a higher frequency than patient-corresponding MSC. When osteogenic and adipogenic commitment of OSDC and MSC were compared, as shown in Figure 3B, osteogenically induced OSDC produced a calcium-containing matrix which was more intensely stained with alizarin red compared to patient-corresponding MSC in osteogenic induction medium. Quantification of bound alizarin red staining showed a 2-fold increase in absorbance in osteogenically differentiated-MSC compared to undifferentiated-MSC culture, while a 5- to 7-fold increase was observed for osteogenically differentiated-OSDC compared to undifferentiated-OSDC cultures. In contrast, adipocyte commitment was more efficient for MSC than for OSDC. As shown in representative images in Figure 3C, following adipogenic differentiation of MSC, more than 50% of cells differentiated into adipocytes, as detected with Nile Red lipid staining, whereas less than 30% of OSDC differentiated toward the adipogenic lineage after adipogenic induction.

Taken together this data shows that compared to MSC, OSDC presented a higher ability to form sphere clones, indicating a potential CSC phenotype, and a lower potential to differentiate towards adipocytes, while they were more efficient at osteogenic differentiation, indicating partial loss of multipotency.

### 2.4. Normal Karyotype or Focused Genetic Aberrations in OSDC Compared to Primary and Secondary OS

**Figure 4:**
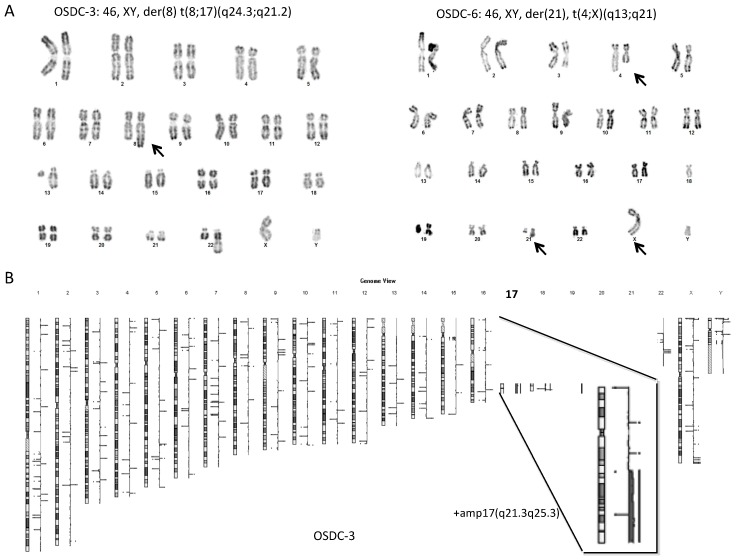
Few chromosomal rearrangements on OSDC-3 and 6. (**A**) Karyotype analysis on OSDC-3 and 6. Abnormal chromosomes are indicated (arrows). Only abnormal chromosomes that were observed in more than 10 metaphases are indicated by arrow; (**B**) Analysis of array comparative genomic hybridization on OSDC-3.

Karyotype analysis was performed on primary cell lines at the earliest possible passage, usually at passage 3 or 4. MSC from all patients (6/6) and 7 out of 9 OSDC showed a normal karyotype (Supplementary Figure S2), with OSDC-3 and 6 presenting a few chromosomal rearrangements (Figure 4A). OSDC-3 displayed an abnormal chromosome 8q with a large additional chromosomal region. OSDC-6 displayed an apparently balanced translocation between the long arms of chromosome 4 and X. Comparative genomic hybridizations analysis (aCGH) were performed to precisely determine the chromosomal imbalances (i.e., deletions and duplications).

**Figure 5:**
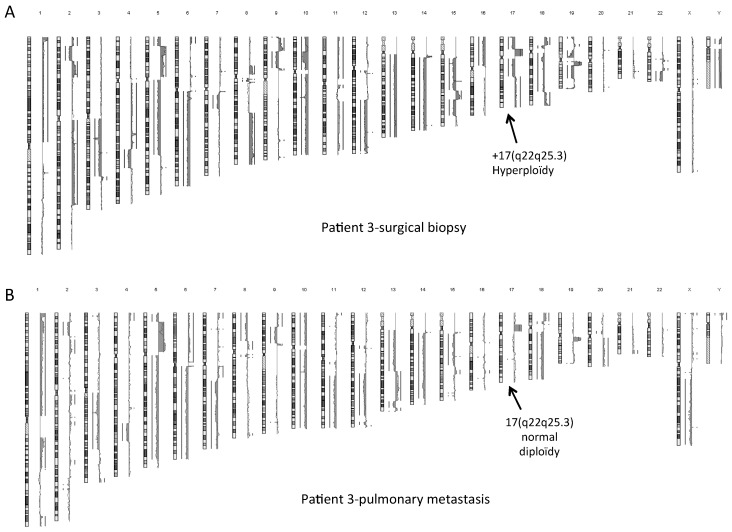
Complex chromosomal rearrangements on tumor samples of patient 3. Analysis of array comparative genomic hybridization on DNA from patient-3, of (**A**) the OS biopsy before poly-chemotherapy and (**B**) of pulmonary metastasis 18 months after the initial treatment. Black arrows show the 17(q22q25.3) hyperploidy that is found in the biopsy sample but not in the metastasis sample.

In OSDC-3, the additional chromosomal region at 8q corresponded to the last 37 Megabases of 17(q21.3q25.3), leading to a duplication of this region (Figure 4B). It was not a constitutional insertion since MSC-3 showed a normal karyotype (Supplementary Figure S2) and a normal aCGH profile. No other relevant large chromosomal abnormalities were detected in OSDC-3. This 17(q22q25) duplication was also identified among the numerous chromosomal abnormalities observed within the pre-treated tumor in the surgical biopsy sample. However, it was not detected within metastasis samples (Figure 5). Further analyses are needed of the 17(q22q25) duplication, which contains 482 genes including *COL1A1, SOX9, RECQL5*, and was described in specific subtypes of genomic changes in breast tumor [28] and rhabdomyosarcoma [29]. 

In OSDC-6, aCGH analysis showed no copy number variations on chromosome 4 and X, confirming the balanced translocation between the long arms of chromosomes 4 and X. We also detected by aCGH a deletion of 1.25 Megabases on 21q including 10 genes among them, *RUNX1* also known as acute myeloid leukemia 1 protein (AML1). We additionally identified at 16p13.1 a cryptic deletion (165 Kilobases) which included a single gene *ERCC4* coding for Excision Repair 4, Endonuclease Catalytic subunit which is involved in germ line mutations [30] and was recently associated with genetic predisposition in sporadic sarcoma [31]. 

Overall, none of the OSDC displayed the numerous chromosome rearrangements usually observed in high grade OS [32]. However, two OSDC, OSDC-3- and 6, presented focused genomic abnormalities whose potential in initiating the cell transformation may be addressed.

### 2.5. OSDC Are Not Tumorigenic but Support OS Progression

All OSDC lines were injected alone in nude and/or SCID mice and, similarly to MSC, they did not induce tumor development after 4 to 6 months of follow-up. Regarding the non-tumorigenicity of OSDC when 1 × 10^6^ cells were injected, we suggest that OSDC are stromal cells of the OS microenvironment rather than potential CSC. 

**Figure 6:**
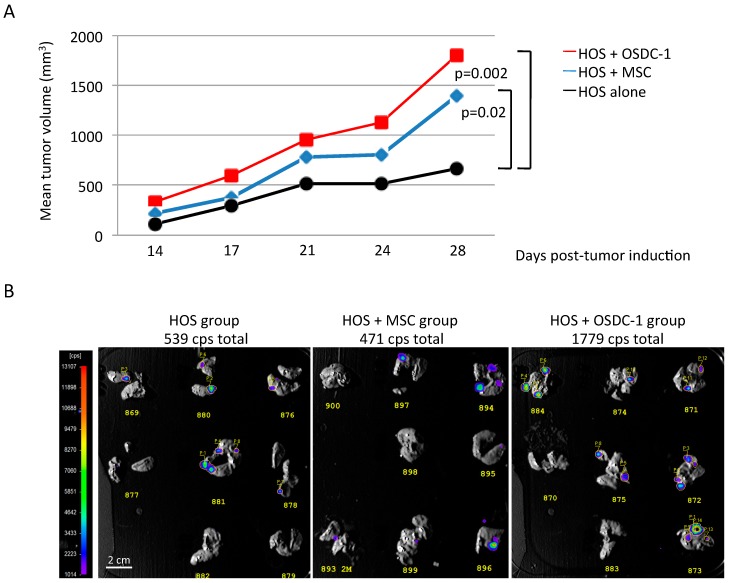
Co-injection of OS-inducing cells (MNNG-HOS cells (HOS)) with OSDC-1 in athymic mouse. (**A**) Mean tumor volumes are presented for three groups of mice (n = 8) injected with 1 million MNNG-HOS cells either alone, or with 0.5 million of MSC or OSDC-1. Significant differences between the MNNG-HOS and the MNNG-HOS + MSC/OSDC-1 groups are indicated. Differences were significant when *p* <0.05. Individual tumor volumes are represented as dots and mean tumor volumes in supplementary Figure S3; (**B**) Images of in vivo bioluminescence detection are shown for excised lung lobes. Luciferase-activity was detected at day 28 after cell injections for each group. Counts per second (cps) of selected areas with value above background were added together as cps total.

In order to observe the effects of OSDC on OS development in nude mice, in the first attempt OSDC-1 were co-injected with Firefly luciferase-expressing MNNG-HOS cells which allow *ex vivo* bioluminescence detection of lung metastases as previously described [33,34]. Tumor volumes and bioluminescence signals were compared to those of mouse groups which have received MNNG-HOS cells alone or combined with MSC derived from bone marrow of healthy patient (Figure 6). As previously observed [35], co-injection of MSC induced a significant increase of MNNG-HOS-induced tumors in comparison with MNNG-HOS cells alone, while total bioluminescence signals from lung metastasis were similar (471 and 539 total counts per second respectively). Concerning the OSDC-1 group, tumor development was faster and lung total bioluminescence was >3-fold higher than those observed for MNNG-HOS alone and MNNG-HOS + MSC groups. However, we observed in each group that bioluminescence was not detected for a few larger nodules despite them being clinically observed in the lungs. Larger nodules had likely progressed into necrotic and fibrotic tissue leading to metabolic changes and loss of luciferase activity as previously demonstrated [34,36]. 

Six additional OSDC were co-injected with MNNG-HOS cells and compared with MNNG-HOS cells injected alone in nude mice (supplementary Figure S3). Only OSDC-4 and 5 induced significant increase of tumor growth, whereas similar tumor growth was observed for OSDC-2, 3, 7, and 9. Regarding the problem of bioluminescence detection for lung nodules noted, nodules were detected by histological analysis. A higher incidence of pulmonary metastasis was detected for MMNG-HOS co-injected with OSDC-2, 3, 4, 5, and 9 compared to injection of MNNG-HOS alone. For OSDC-7, metastasis rate was similar (Table 4).

As OSDC-1 was the more competent to induce tumor progression, paracrine factors in the conditioned medium (CM) of OSDC-1 were compared to those in the CM of bone marrow MSC from healthy donors (Supplementary Figure S4). MSC and OSDC-1 showed similar interleukins (IL) secretion levels, particularly IL-6 and IL-10. However, IL-7 and IL-15 secretion levels by OSDC-1 were undetectable, contrarily to MSC. Interferon-γ (IFN-γ), bFGF, and Regulated on Activation, Normal T Cell Expressed and Secreted (RANTES alias CCL5) were secreted at higher levels by OSDC-1 compared to MSC, while Vascular Endothelial Growth Factor (VEGF) secretion was 4 times higher by MSC compared to OSDC-1. 

In conclusion, OSDC lines did not induce OS alone in SCID mice, but 3 out of 7, and 6 out of 7, showed a supportive activity on local tumor development, or on metastatic progression to lungs, respectively, when they were co-injected with OS cells in nude mice. This supportive activity could be supported by RANTES (CCL5).

## 3. Discussion

High-grade osteosarcoma is characterized by a relative high treatment failure and metastasis development. This is confirmed in our study, with less than half of the patients achieving event free survival. Interestingly, only half of the patients whose tumor had a good response to chemotherapy were event free during the follow up. In spite of the small cohort presented in this study, this observation needs further exploration. Indeed, remaining viable tumor cells after pre-operative chemotherapy is one of the most important prognosis factors known [37]. Taking into consideration our results, extensive explorations on larger cohorts are warranted. 

Currently, the majority of studies aiming to gain an understanding of the underlying mechanisms of OS employ OS cell lines. However, the majority of those cell lines are rather old, some of them being isolated as far back as the 1970s [38]. Hence some clones possessing enhanced growth capacities in flasks might have been selected by this extended cell culture. As a consequence, study material might be far removed from the tumor reality. In order to reduce this gap, we committed to acquiring data from cells which reflect as close as possible the cells in the native tumor, by working with freshly isolated tumor derived cells. In addition, as OS is likely to derive from MSC, we sought to compare OSDC to bone marrow derived MSC from the same patient, which increases the originality of our work. Furthermore, we cultured OSDC and MSC in parallel under the same conditions in order to achieve directly comparable insights. No specific markers for OS cells and OS CSC currently exist or are identified in the present study. Rather, we found OSDC and MSC to be very similar. However, tumor cells outside of the tumor and host microenvironment might be more vulnerable and CSC could disappear after a few cell culture passages. Using single cell analysis at early stages after cell isolation could have aided in this characterization, however amplification from a single cell would not have permitted the expansion of cells in adequate numbers for all the in vitro and in vivo assessments carried out in the current study. 

Cell culture with semi solid and anchorage-independent conditions were used to isolate potential OS-CSC in several previous studies [13], and only one team has presented markers to identify OS-CSC [24]. In the current study, we used anchorage-independent cell culture conditions to isolate potential OS-CSC from fresh tumors. OSDC sphere clones were obtained for all OSDC populations but one, while conversely almost no clones were obtained from MSC. OSDC sphere clones were obtained with a lower frequency than that observed by Gibbs et al. with OS biopsy [24], with perhaps different media preparations employed being a contributing factor to this observed difference. Nevertheless, OSDC showed growth capacities in semi solid and anchorage independent conditions. We did not observe OSDC tumorigenic capacities in vivo*,* however it is important to note that only the OSDC-5 population was obtained before chemotherapy (the other eight being obtained from tumor resection specimens) and this patient OSDC showed the lowest rate of sphere rate formation (OSDC-3 excluded). As CSC could be involved in local recurrence and metastasis [17], chemotherapy might have selected CSC with better ability to grow in anchorage independent condition. However, our observations may need to be confirmed in a larger cohort of cases. 

We did not use stemness surface marker to select CSC given that a switch of phenotype toward stemness may be induced by anchorage-independent conditions. Indeed, in previous work, we observed that the OS cell line MNNG-HOS which is not CSC, over expressed stemness markers when cultured in spheres compared to monolayer culture conditions (*OCT4* (>200×), *SOX2* (>50×), and *NANOG* (>9×)) [33]. Differentiation potential analysis showed increased OSDC osteoblastic capacities compared to MSC and less adipogenic differentiation capacities. Thus, OSDC were osteoblastic committed cells with a CSC phenotype. This OSDC osteoblastic commitment did not seem to be present in Gibbs’ work [24]. We also studied chondrogenic differentiation capacities, however we did not have cells in sufficient numbers to achieve comparative experiments. Furthermore, OSDC did not show tumorigenic capacities in an immunocompromised mouse model. 

OS is a complex genetic disease. In our study, OSDC presented cytogenetic abnormalities in two cases, whereas MSC did not show any. Conventional OS is characterized by highly complex numerical and structural chromosomal abnormalities. Thus, cytogenetic abnormalities observed in OSDC-3 and 6 did not correspond to usual OS cytogenetic abnormalities. Interestingly, one of the observed abnormalities corresponded to *ERCC4* gene locus deletion. ERCC4 is an enzyme involved in nucleotide excision-repair DNA damages and is deleted in Fanconi anemia, which is a genomic instability disorder [39]. This deletion could be hence a pre-tumoral abnormality consistent with a chromothripsis hypothesis. In the second case, the 17(q22q25) region contains *SOX9* and *COL1A1* which are involved in chondroblastic and osteoblastic differentiation pathways. Moreover, interestingly, 17(q22q25) amplification is linked with increased aggressiveness in breast cancer tumors [28]. None of these observed abnormalities were reported by Kovac et al. who described 12 OS driver genes [40] that interestingly did not include *P53*. The *P53* mutation has been described as a key feature in cancerogenesis. However, its mutation does not seem to be central in human OS, since it has been found to be mutated in only 38% of OS by Overholtzer et al. [41]. In addition, patients suffering from Li Fraumeni syndrome (*P53* congenital mutation) do not systematically develop OS, and rather have a 12% risk of developing OS [42]. Regarding the OSDC-3 17(q22q25) duplication, interestingly, it was found in the biopsy sample from patient-3, within multiple cytogenetic abnormalities, but neither in the tumor resection sample from which OSDC were isolated, nor within two distinct metastases. This indicates first that this duplication may not be responsible for the OS development. Secondly, since OSDC could likely be a small subpopulation within the tumor, aCGH may not have sorted it out. In vitro culture could have selected and amplified OSDC, rendering this duplication detectable and may explain why this duplication was found in OSDC and not in the tumor from which OSDC derived (i.e., tumor sample after neo-adjuvant chemotherapy). 

Finally, this suggests that although OSCD did not initiate tumor growth in vivo, they could be pre tumor cells. Indeed, Hochane et al. showed that human MSC with four oncogenic hits were not tumorigenic however their oncogenic potential was revealed by stress cell culturing using pesticides [43]. In our study, an attempt was made to stress OSDC by the use of culture medium containing Tumor Necrosis Factor-α and IFN-γ, as reported by Wang et al. [44]. This led to significant cell growth arrest and increased cell death, resulting in cell stocks of insufficient quantity to perform the desired in vitro and in vivo experiments. Furthermore, tumor potential assessed by in vivo tumor growth is questionable. Indeed, some human OS cell lines e.g., HOS did not give rise to tumors in vivo unless they were transformed by a mutagenic agent [45]. Moreover, OS cells need growth support cells which can be possibly species specific. Currently, animal models, primarily mouse models are used to test tumorigenicity in vivo, but since there are many differences between the mouse and human species, some authentic CSC might not be discovered by these xenogenic testing models [46]. 

OSDC and MSC co-injections with tumor cells led to increased tumor growth and eventually to metastases. Despite 5 OSDC lines from patients with lung metastatic progression demonstrating pro-metastatic supportive activity in mice, we cannot conclude a correlation between the two observations since we only included one patient with no metastatic progression in this in vivo study. However, from a histological point, both OSDC and MSC were only transiently detected within the tumor (not detected after 10 days injection). The in vivo effects of co-injected OSDC/MSC may be primarily due to paracrine factors secreted, as is observed in regenerative medicine including cell therapy for bone repair [33,47]. MSC may facilitate extravasation of tumor cells, which is known to be partly mediated through secreted chemokines (i.e., C-X-C motif chemokine 12 (CXCL12)) [48,49]. After MSC recruitment by tumors, pro tumor effects could be linked to RANTES (CCL5) [50]. Interestingly, OSDC-1 secreted elevated levels of RANTES compared to MSC in our study. Cells that support tumor progression have been reported in various different cancers, in carcinoma in particular, and were referred to as cancer associated fibroblasts. For systematic characterization, we used common markers of CAF [51,52], even if OS is not an epithelial cancer. Vimentine and ASMA were expressed both by OSDC and MSC. However, we cannot consider OSDC to be similar to fibroblasts. Rather, OSDC were very morphologically and phenotypically similar to MSC. They possessed multipotent differentiation capacities, albeit to varying degrees. Minor genetic modifications were observed for two OSDC populations, which could be assigned to the tumor influence on the microenvironment. Indeed, genetic modifications of MSC in the tumor microenvironment have been reported in hematologic diseases like leukemia, with various structural and/or numerical chromosome aberration [53] and carcinoma, e.g., breast cancer, with *P53*, *PTEN,* and *WFDC1* mutations [54].

MSC aging was shown to be associated with an increase in senescence level and a decrease in osteogenic differentiation potential [55]. In our study, we observed the earliest appearance of senescence in OSDC derived from the oldest patient (patient 6, 36-years old), however, it was not associated with a decrease in osteogenic compared to adipogenic commitment. Aging and senescence of MSC/OSDC may modulate OS progression, however it was not possible to amplify OSDC-6 in sufficient numbers, due to early senescence in culture (passage 8), in order to test their effect during co-injection with OS cells in nude mice.

Tumor microenvironment is a key point in any tumor development. For example, it has been shown that the presence of CD163 positive macrophages and CD8-positive cytotoxic lymphocytes in the OS microenvironment was associated with a better overall survival [56]. In our study, we did not test the immunomodulatory effect of MSC/OSDC, while stromal cells may inhibit T lymphocyte activity against tumor cells [57].

Concerning the stromal component, Brune et al. suggested that OSDC are microenvironment MSC, and observed that they are significantly increased in number in the OS microenvironment, about 1000 times more compared to bone marrow or adipose tissue [13]. Those cells could have various different origins: the bone growth plate which is close to the tumor; or as MSC have migration capacities they could also come from blood vessel walls or migrate from other tissues through the blood circulation [58]. After MSC recruitment by the tumor, an MSC pro tumor effect could be linked to RANTES (CCL5) [50]. This could be an interesting connection given that high *CCL5* expression in OS is associated with a bad prognosis [59] and that CCL5 secretion was elevated by OSDC-1 compared to MSC in our study.

## 4. Materials and Methods

### 4.1. Ethics Approval Statement

Osteosarcoma samples and bone marrow aspirates were obtained from patients during orthopedic surgical procedures in Tours University Hospital (Tours, France). Written consent was obtained from informed patients in accordance with French law (Art. L. 1245-2 of the French public health code, Law No. 2004-800 of 6 August 2004, Official Journal of 7 August 2004).

Experiments involving mice were conducted in accordance with French guidelines (named “Charte nationale portant sur l’éthique de l’expérimentation animale” by the French ethics committee) and were approved by the regional committee on animal ethics (CEEA.PdL.06) with project authorization number 2013.4 (approval date: 24 January 2013). Welfare of the animals was checked daily by observing their behavior and their body weight was measured once per week. The mice were anesthetized by inhalation of an isoflurane-air mix (4%, 1 L/min) before any surgical manipulation and all efforts were made to minimize suffering.

### 4.2. Primary Cell Cultures

OS samples were obtained from resected specimens, after chemotherapy for all patients, except OSDC-5. A small piece of approximately 1 cm^3^ was harvested, fragmented, and stored under sterile conditions. Storage conditions are indicated in Table 2. The sample was only minced for patient 1, while other samples were treated with enzymatic dissociation buffer containing collagenase I (200 U/mL) or in an optimized mix for high yield of tumor cells and non-tumor cells combined with the preservation of cell surface epitopes (MACS human tumor dissociation kit, Miltenyi Biotec, Paris, France). After at least 1 h at 37 °C, the cell suspension was filtered through a nylon mesh (70 µm), washed by centrifugation, and red blood cells were lysed before counting. Extracted cells were seeded in tissue culture treated flasks and under semi solid anchorage-independent culture condition (4200 cells per cm^2^).

OSDC and MSC were cultured in Minimum Essential Medium alpha (α-MEM) with nucleosides and 1 g/L d-Glucose (Gibco^®^ MEM α; Life technologies, Saint Aubin, France) and supplemented with 1% antibiotics (Penicillin 100 U/mL and Streptomycin 100 mg/L; Invitrogen, Cergy-Pontoise, France), 10% fetal bovine serum (FBS), Dominique Dutscher, Brumath, France), and 1 ng/mL basic-Fibroblast Growth Factor (bFGF), at 37 °C in a humidified atmosphere (5% CO_2_/95% air). The cells were harvested using trypsin (0.5 g/L) ethylene-diamine-tetraacetic acid (EDTA) (0.2 g/L) (Cambrex Bio Sciences, Verviers, Belgium).

For culture under anchorage-independent conditions, medium (α-MEM 10% FBS, 1% antibiotics, and 1 ng/mL bFGF) was supplemented with 1.05% of methylcellulose (R&D Systems, Lille, France) and 4 × 10^4^ OSDC or MSC were seeded per well in 6-well low adherent culture plates (low attachment plate, Corning Corp, Boston, MA, USA). Spheres containing more than 20 cells were selected under a microscope and mechanically dissociated before counting and seeding on treated culture polystyrene flasks (Corning, Boston, MA, USA).

MSC were derived from iliac crest aspirates as previously described [60]. After prior written consent, bone marrow was harvested from the iliac crest of healthy donors during total hip replacement procedures. Bone marrow was also harvested from the iliac crest of osteosarcoma patients during biopsy procedures. 10 mL bone marrow was harvested by using a Jamshidi trepan with a heparinized syringe. Briefly, after MSC isolation, 2.5 × 10^4^ cells per cm^2^ were seeded in tissue culture treated flasks with α-MEM 10% FBS, 1% antibiotics, 1 ng/mL bFGF. The expansion medium was renewed every 3–4 days until adherent cells reached 90% confluency, corresponding to a 10-fold increase in cell number. Then, cells were harvested using trypsin/EDTA solution.

Following passage 1, OSDC and MSC were seeded at 2500 cells per cm^2^ at each passage. They were amplified up to passage 3 for flow cytometry analysis, sphere assay, and differentiation assays and up to passage 4 or 5 for in vivo assays.

### 4.3. Flow Cytometry Analysis

Briefly, 1 × 10^5^ cells at passage 3 or 4 were stained for 15 min in 100 μL phosphate-buffered saline with fluorescein isothiocyanate (FITC)- or phycoerythrin (PE)-conjugated antibodies to detect CD34, CD45, CD44, CD73, CD90, or CD105. Antibodies were sourced from BD Biosciences (Le Pont de Claix, France), except CD105 which was obtained from BioLegend through Ozyme (Paris, France). The ratio of mean fluorescent intensity (MFI) was defined as the ratio of MFI obtained with one antigen-surface marker to the MFI obtained with the corresponding isotype (IgG-FITC or IgG-PE). Fluorescent intensities of 50,000 events were acquired using a FC500 flow cytometer and CXP software (version 2.2, Beckman Coulter, Villepinte, France).

### 4.4. Fluorescent Microscopy Analysis

For immunodetection of ASMA, 1 × 10^4^ cells per cm^2^ were seeded on Nunc Lab-Tek Chambered Coverglass (Thermo Scientific, Waltham, MA, USA) and cultured until 50% confluency. Cells were rinsed with PBS, fixed in 4% paraformaldehyde for 20 min at room temperature (RT), rinsed in PBS, and stored at 4 °C. Cells were permeabilized for 30 min at RT with 0.05% Triton X-100 in PBS complemented with 1% Bovine Serum Albumine (BSA) and 1% Normal Goat Serum (NGS) to reduce non-specific staining. Incubation with an antibody directed against ASMA (MAB1420; R&D System) at a dilution of 1/100 in PBS with 1% BSA and 1% NGS was performed overnight at 4 °C. Cells were washed three times in PBS, followed by incubation with Alexafluor 488 conjugated goat-anti-mouse IgG (Life Technologies, Saint-Aubin, France) at a dilution of 1/500 for 1 h at RT in darkness. After several washes, nuclei were stained with 4′,6-Diamidino-2-Phenylindole, Dihydrochloride (DAPI; Life Technologies) at a concentration of 1/40,000 for 10 min at RT in darkness. Images were captured using a Nikon A1RSi confocal laser-scanning microscope (Nis Elements Confocal, Nikon, Amstelveen, The Netherlands).

### 4.5. Osteogenic and Adipogenic Differentiation 

To induce differentiation towards osteogenic and adipogenic lineages, cells were cultured in an Osteogenesis Medium (OM: α-MEM, 2% FBS, 1 mM NaH2PO4, 0.05 mM ascorbic acid, 10^−7^ M dexamethasone) for 21 days or in an adipogenesis medium (α-MEM, 10% FBS, 10^−6^ M dexamethasone, 60 µM indometacin, 0.5 mM 3-isobuthyl-1-methylxanthine) for 14 days, respectively. Mineralization was observed by alizarin red staining and the bound alizarin red stain was quantified as previously described [61]. Lipid droplets were stained with Nile Red (Sigma, Dorset, UK) and nuclei were stained with DAPI.

### 4.6. Genetic Analysis

Karyotyping based on R or G banding was performed using standard methods on metaphase spreads from primary cell lines, MSC and OSDC of the patients. 

Genomic DNAs were extracted from primary cell lines, MSC, and OSDC using standard protocols. Array comparative genomic hybridization (aCGH) experiments were performed by using Agilent Human Genome CGH 400K oligonucleotide arrays or 60K oligonucleotide arrays with the ISCA design (Agilent, Santa Clara, CA, USA; Available online: www.agilent.com) following the protocols provided by Agilent. Arrays were scanned with G2565CA Microarray Scanner System and analyzed with CytoGenomics v3.0.6.6 (Agilent Technologies, Santa Clara, CA, USA).

### 4.7. Cell Senescence Analysis

X gal staining was performed to show beta-galactosidase activity which is activated during cell senescence. 

### 4.8. Condition Medium and Multiplex Analysis

OSDC-1 and healthy bone marrow MSC were cultured to near confluence with alpha-MEM medium supplemented with 10% FBS, washed twice, and cultured overnight in serum-free medium which was then collected and frozen (−20 °C), constituting OSDC-1 or MSC-conditioned medium.

Quantification of soluble factors was performed using the Luminex technology (Bio-Plex Pro Assays, Bio-Rad, Marnes la Coquette, France) according to the manufacturer’s instructions. Interleukins (IL), chemokine ligands (CCL and CXCL), growth factors (fibroblast GF-2, vascular endothelial GF-A, platelet-derived GF-ββ, tumor necrosis factor-α, interferon-γ, leukemia inhibitory factor) and adipokines (leptin, resistin, visfatin) were measured.

### 4.9. MNNG-HOS-Induced Osteosarcoma in Nude Mice 

As previously described [33], MNNG-HOS cells (American Type Culture Collection CRL-1547) were used to induce a primary tumor against the periosteum, behind the tibialis anterior muscle, of four-week-old female athymic mice (NMRI nu/nu; Elevages Janvier, Le Genest St Isle, France). They were injected with 1 × 10^6^ MNNG-HOS cells alone, or with 5 × 10^5^ OSDC or MSC in 50 μL PBS. The tumor volume (mm^3^) was calculated with the formula (l^2^ × L)/2 where l and L represent the smallest and the largest diameters (mm) respectively, measured by using a vernier caliper. Luciferase-expressing MNNG-HOS cells were obtained using lentiviral production and transduction as described by Rousseau et al. and they were injected in vivo as described above [34]. On the basis of 25  g weight, mice were intraperitoneally injected with 250 μl of a Rompun-Ketalar solution (8% and 13%, respectively, in PBS) before intraperitoneal injection of 3 mg d-luciferin (Interchim, Montluçon, France) dissolved in 250 μL of water. After 7 min, mice were sacrificed for quick extraction of the lungs, which were placed into a photon Imager NightOWL LB 981 (Berthold technologies, Thoiry, France). Bioluminescence acquisition was performed twice for 1.5 min each. Photons released from luciferin degradation were measured as counts per second (cps) per selected area.

### 4.10. Histology Analysis

Lungs were fixed in 10% buffered formaldehyde. After embedding in paraffin wax, the samples were sectioned and stained with haematoxylin–eosin solution. The presence of tumor nodules in the lungs was determined for each animal by assessing a minimum of three sections (5 μm-thick) per explant, at a minimum of 500 µm thickness spacing between sections.

### 4.11. Statistical Analysis

GraphPadInStat v3.02 software (La Jolla, CA, USA) was used. Kruskal-Wallis and Wilcoxon rank-sum non-parametric tests were used for analysis of in vivo results. Significant difference was assessed with a p value less than 0.05. 

## 5. Conclusions

Osteosarcoma derived cells are likely to be cancer associated MSC, committed towards the osteoblastic lineage, with probable tumor induced changes e.g., genetic abnormalities, anchorage independent growing capacities. Thus, MSC show their wide implications: in regenerative medicine, bone consolidation, bone reconstruction, and tumor growth. Given that MSC have been described as an OS metabolic modulator, exploring potential metabolic modifications could also help to better characterize OSDC. Ultimately, individualization of specific markers of OS microenvironment could lead us to find targeted drug therapies.

## Figures and Tables

**Table 1:** Clinical characteristics of high-grade osteosarcoma (OS) patients.

Patient	1	2	3	4	5	6	7	8	9
Year of diagnosis	2007	2010	2010	2014	2014	2015	2015	2015	2015
Age	21	18	16	23	30	36	18	14	14
Gender	F	M	M	M	F	M	M	M	M
Histological subtype	F	OB, CH, F	OB	OB	CH	OB, CH	OB, CH, F	OB, T	OB
Tumor site	proximal humerus	Distal femur	Distal femur	Distal tibia	pelvis	Distal femur	proximal tibia	proximal fibula	proximal fibula
Limb salvage	Yes	No	Yes	No	Yes	Yes	Yes	Yes	Yes
Metastasis at diagnosis	Yes	No	No	No	No	No	Yes	No	n.d.
Response to NAPC	GR	PR	GR	PR	PR	GR	GR	PR	PR
% residual tumor cells	6%	55%	7%	20%	30%	8%	<1%	11%	52%
Local recurrence	No	No	No	No	No	No	No	No	No
Metastasis progression	Yes	Yes	Yes	Yes	Yes	No	No	No	No
site	lung	lung	lung	lung	lung				
Outcomes	DOD	DOD	AWD	DOD	AWD	NED	NED	NED	NED

Gender: male (M), female (F). Histological subtypes: osteoblastic (OB), chondroblastic (CH), fibroblastic (F), telangiectatic (T). Good responder (GR) or poor responder (PR) to neo-adjuvant poly-chemotherapy (NAPC): GR is defined as less than 10% alive residual tumor cells on tumor sample resection after NAPC. More than 10% residual alive tumor cells on tumor sample resection define PR. Patient outcomes: dead of disease (DOD), alive with disease (AWD), alive with no evidence of disease (NED). Not determined (n.d.).

**Table 2:** Description of primary cell lines derived from OS samples (OSDC) and from bone marrow (MSC).

**Patient’s OSDC**	**1**	**2**	**3**	**4**	**5**	**6**	**7**	**8**	**9**
Time of tumor sample collection for primary cell lines	post-NAPC	post-NAPC	post-NAPC	post-NAPC	pre-NAPC	post-NAPC	post-NAPC	post-NAPC	post-NAPC
Sample storage before dissociation	in culture medium P/S at 4 °C for 12 h			in SVF with 10% DMSO at −80 °C for 1 to 4 weeks			in MACS Tissue Storage Solution at 4 °C for 24 to 72 h		
Enzymatic dissociation	No	in Collagenase I (InVitrogen) during 1 to 4 h at 37 °C					in MACS human tumor dissociation kit (Miltenyi Biotech) for 1 h at 37 °C		
Sphere rate (10^−5^)	n.d.	65	0	3.3	0.67	5.5	15.8	n.d.	47
**Patient’s MSC**	**n.a.**	**2**	**3**	**4**	**5**	**6**	**7**	**n.a.**	**n.a.**
Time of BM aspirates	n.a.	pre-NAPC	post-NAPC	post-NAPC	pre-NAPC	pre-NAPC	pre-NAPC	n.a.	n.a.
Sphere rate (10^−5^)	n.a.	0	0.13	n.a.	n.a.	0	0	n.a.	n.a.

Adherent cells were derived from OS samples (OSDC) or from bone marrow (MSC) when available (not available n.a.). Tissue samples were collected before or after neo-adjuvant poly-chemotherapy (pre- or post-NAPC). Sample storage conditions and dissociation method are indicated. Sphere rates obtained following anchorage-independent conditions are indicated when determined. Not determined (n.d.). Not available (n.a.).

**Table 3:** Ratios of mean fluorescent intensity for cluster of differentiation (CD) antigens on primary cell lines derived from OS samples (OSDC) and from bone marrow (MSC).

	9 AD-OSDC		6 AD-MSC		6 SP-OSDC	
	Mean	SEM	Mean	SEM	Mean	SEM
CD34	1	0	1	0	2	1
CD45	1	0	1	1	1	0
CD44	128	99	144	100	210	34
CD73	44	32	62	41	91	17
CD90	274	224	208	164	474	65
CD105	81	35	86	56	51	14

Ratio of mean fluorescent intensity and standard error of the mean (SEM) are indicated for primary cell lines isolated by adherence (AD) on tissue culture treated plastic or obtained in sphere under anchorage-independent culture conditions (SP).

**Table 4:** Incidence of pulmonary metastasis in nude mice.

Patient’s OSDC	1	2	3	4	5	7	9
HOS alone	5/8	2/8	2/8	5/8	5/8	6/8	1/8
HOS + OSDC	6/8	5/8	5/8	7/8	7/8	6/8	2/8

Number of mice bearing lung metastasis is indicated for a total of 8 mice in each group. Osteosarcoma derived cells (OSDC) were derived from OS of patients 1–5 and 7. OS was induced using MNNG-HOS (HOS) cells alone or combined with OSDC at a ratio of 2:1, which were injected against the tibia of athymic mice. Lung metastases were detected following hematoxylin–eosin staining on three histological sections per lung.

## Supplementary Materials

Supplementary materials can be found at http://www.mdpi.com/1422-0067/19/3/707/s1.

## Abbreviations

OS	Osteosarcoma
OSDC	Osteosarcoma Derived Cells
MSC	Mesenchymal Stem Cells
CSC	Cancer Stem Cells
CAF	Cancer associated fibroblast

## References

[1] Dorfman H.D., Czerniak B. (1995). Bone cancers. Cancer.

[2] Rosenberg E., Cleton-Jansen A.-M., de Pinieux G., Fletcher C.D.M., Bridge J.A., Hogendoorn P.C.W., Mertens F. (2013). Conventional osteosarcoma. WHO Classification of Tumours of Soft Tissue and Bone.

[3] Ottaviani G., Jaffe N. (2009). The epidemiology of osteosarcoma. Cancer Treat. Res..

[4] Mialou V., Philip T., Kalifa C., Perol D., Gentet J.-C., Marec-Berard P., Pacquement H., Chastagner P., Defaschelles A.-S., Hartmann O. (2005). Metastatic osteosarcoma at diagnosis: Prognostic factors and long-term outcome—The French pediatric experience. Cancer.

[5] Anninga J.K., Gelderblom H., Fiocco M., Kroep J.R., Taminiau A.H.M., Hogendoorn P.C.W., Egeler R.M. (2011). Chemotherapeutic adjuvant treatment for osteosarcoma: Where do we stand?. Eur. J. Cancer.

[6] Longhi A., Errani C., de Paolis M., Mercuri M., Bacci G. (2006). Primary bone osteosarcoma in the pediatric age: State of the art. Cancer Treat. Rev..

[7] Ferrari S., Palmerini E. (2007). Adjuvant and neoadjuvant combination chemotherapy for osteogenic sarcoma. Curr. Opin. Oncol..

[8] Allison D.C., Carney S.C., Ahlmann E.R., Hendifar A., Chawla S., Fedenko A., Angeles C., Menendez L.R. (2012). A meta-analysis of osteosarcoma outcomes in the modern medical era. Sarcoma.

[9] Trama A., Botta L., Foschi R., Ferrari A., Stiller C., Desandes E., Maule M.M., Merletti F., Gatta G. (2016). Survival of European adolescents and young adults diagnosed with cancer in 2000–07: Population-based data from EUROCARE-5. Lancet Oncol..

[10] Dahlin D.C., Coventry M.B. (1967). Osteogenic sarcoma. A study of six hundred cases. J. Bone Joint Surg. Am..

[11] Martin J.W., Squire J.A., Zielenska M. (2012). The Genetics of Osteosarcoma. Sarcoma.

[12] Gillette J.M., Gibbs C.P., Nielsen-Preiss S.M. (2008). Establishment and characterization of OS 99-1, a cell line derived from a highly aggressive primary human osteosarcoma. In Vitro Cell. Dev. Biol. Anim..

[13] Brune J.C., Tormin A., Johansson M.C., Rissler P., Brosjö O., Löfvenberg R., von Steyern F.V., Mertens F., Rydholm A., Scheding S. (2011). Mesenchymal stromal cells from primary osteosarcoma are non-malignant and strikingly similar to their bone marrow counterparts. Int. J. Cancer.

[14] Arndt C.A., Crist W.M. (1999). Common musculoskeletal tumors of childhood and adolescence. N. Engl. J. Med..

[15] Dujardin F., Binh M.B.N., Bouvier C., Gomez-Brouchet A., Larousserie F., Muret A., de Louis-Brennetot C., Aurias A., Coindre J.-M., Guillou L. (2011). MDM2 and CDK4 immunohistochemistry is a valuable tool in the differential diagnosis of low-grade osteosarcomas and other primary fibro-osseous lesions of the bone. Mod. Pathol..

[16] Forment J.V., Kaidi A., Jackson S.P. (2012). Chromothripsis and cancer: Causes and consequences of chromosome shattering. Nat. Rev. Cancer.

[17] Basu-Roy U., Basilico C., Mansukhani A. (2013). Perspectives on cancer stem cells in osteosarcoma. Cancer Lett..

[18] Abarrategi A., Tornin J., Martinez-Cruzado L., Hamilton A., Martinez-Campos E., Rodrigo J.P., González M.V., Baldini N., Garcia-Castro J., Rodriguez R. (2016). Osteosarcoma: Cells-of-Origin, Cancer Stem Cells, and Targeted Therapies. Stem Cells Int..

[19] Cleton-Jansen A.-M., Anninga J.K., Briaire de Bruijn I.H., Romeo S., Oosting J., Egeler R.M., Gelderblom H., Taminiau A.H.M., Hogendoorn P.C.W. (2009). Profiling of high-grade central osteosarcoma and its putative progenitor cells identifies tumourigenic pathways. Br. J. Cancer.

[20] Deschaseaux F., Pontikoglou C., Sensébé L. (2010). Bone regeneration: The stem/progenitor cells point of view. J. Cell. Mol. Med..

[21] Xiao W., Mohseny A.B., Hogendoorn P.C.W., Cleton-Jansen A.-M. (2013). Mesenchymal stem cell transformation and sarcoma genesis. Clin. Sarcoma Res..

[22] Wang J.-Y., Wu P.-K., Chen P.C.-H., Lee C.-W., Chen W.-M., Hung S.-C. (2017). Generation of Osteosarcomas from a Combination of Rb Silencing and c-Myc Overexpression in Human Mesenchymal Stem Cells. Stem Cells Transl. Med..

[23] Skoda J., Nunukova A., Loja T., Zambo I., Neradil J., Mudry P., Zitterbart K., Hermanova M., Hampl A., Sterba J. (2016). Cancer stem cell markers in pediatric sarcomas: Sox2 is associated with tumorigenicity in immunodeficient mice. Tumor Biol..

[24] Gibbs C.P., Kukekov V.G., Reith J.D., Tchigrinova O., Suslov O.N., Scott E.W., Ghivizzani S.C., Ignatova T.N., Steindler D.A. (2005). Stem-Like Cells in Bone Sarcomas: Implications for Tumorigenesis. Neoplasia.

[25] Adhikari A.S., Agarwal N., Wood B.M., Porretta C., Ruiz B., Pochampally R.R., Iwakuma T. (2010). CD117 and Stro-1 Identify Osteosarcoma Tumor-Initiating Cells Associated with Metastasis and Drug Resistance. Cancer Res..

[26] Buchsbaum R.J., Oh S.Y. (2016). Breast Cancer-Associated Fibroblasts: Where We Are and Where We Need to Go. Cancers.

[27] Dominici M., Le Blanc K., Mueller I., Slaper-Cortenbach I., Marini F., Krause D., Deans R., Keating A., Prockop D., Horwitz E. (2006). Minimal criteria for defining multipotent mesenchymal stromal cells. The International Society for Cellular Therapy position statement. Cytotherapy.

[28] Melchor L., Alvarez S., Honrado E., Palacios J., Barroso A., Díez O., Osorio A., Benítez J. (2005). The accumulation of specific amplifications characterizes two different genomic pathways of evolution of familial breast tumors. Clin. Cancer Res..

[29] Pandita A., Zielenska M., Thorner P., Bayani J., Godbout R., Greenberg M., Squire J.A. (1999). Application of comparative genomic hybridization, spectral karyotyping, and microarray analysis in the identification of subtype-specific patterns of genomic changes in rhabdomyosarcoma. Neoplasia.

[30] Manandhar M., Boulware K.S., Wood R.D. (2015). The ERCC1 and ERCC4 (XPF) genes and gene products. Gene.

[31] Chan S.H., Lim W.K., Ishak N.D.B., Li S.-T., Goh W.L., Tan G.S., Lim K.H., Teo M., Young C.N.C., Malik S. (2017). Germline Mutations in Cancer Predisposition Genes are Frequent in Sporadic Sarcomas. Sci. Rep..

[32] Bayani J., Zielenska M., Pandita A., Al-Romaih K., Karaskova J., Harrison K., Bridge J.A., Sorensen P., Thorner P., Squire J.A. (2003). Spectral karyotyping identifies recurrent complex rearrangements of chromosomes 8, 17, and 20 in osteosarcomas. Genes. Chromosomes Cancer.

[33] Avril P., Le Nail L.-R., Brennan M.Á., Rosset P., De Pinieux G., Layrolle P., Heymann D., Perrot P., Trichet V. (2016). Mesenchymal stem cells increase proliferation but do not change quiescent state of osteosarcoma cells: Potential implications according to the tumor resection status. J. Bone Oncol..

[34] Rousseau J., Escriou V., Perrot P., Picarda G., Charrier C., Scherman D., Heymann D., Rédini F., Trichet V. (2010). Advantages of bioluminescence imaging to follow siRNA or chemotherapeutic treatments in osteosarcoma preclinical models. Cancer Gene Ther..

[35] Perrot P., Rousseau J., Bouffaut A.-L., Rédini F., Cassagnau E., Deschaseaux F., Heymann M.-F., Heymann D., Duteille F., Trichet V. (2010). Safety Concern between Autologous Fat Graft, Mesenchymal Stem Cell and Osteosarcoma Recurrence. PLoS ONE.

[36] Brullé L., Vandamme M., Riès D., Martel E., Robert E., Lerondel S., Trichet V., Richard S., Pouvesle J.-M., Le Pape A. (2012). Effects of a Non Thermal Plasma Treatment Alone or in Combination with Gemcitabine in a MIA PaCa2-luc Orthotopic Pancreatic Carcinoma Model. PLoS ONE.

[37] Hauben E.I., Weeden S., Pringle J., van Marck E.A., Hogendoorn P.C.W. (2002). Does the histological subtype of high-grade central osteosarcoma influence the response to treatment with chemotherapy and does it affect overall survival? A study on 570 patients of two consecutive trials of the European Osteosarcoma Intergroup. Eur. J. Cancer.

[38] Billiau A., Edy V.G., Heremans H., Van Damme J., Desmyter J., Georgiades J.A., De Somer P. (1977). Human interferon: Mass production in a newly established cell line, MG-63. Antimicrob. Agents Chemother..

[39] Mori T., Yousefzadeh M.J., Faridounnia M., Chong J.X., Hisama F.M., Hudgins L., Mercado G., Wade E.A., Barghouthy A.S., Lee L. (2018). ERCC4 variants identified in a cohort of patients with segmental progeroid syndromes. Hum. Mutat..

[40] Kovac M., Blattmann C., Ribi S., Smida J., Mueller N.S., Engert F., Castro-Giner F., Weischenfeldt J., Kovacova M., Krieg A. (2015). Exome sequencing of osteosarcoma reveals mutation signatures reminiscent of BRCA deficiency. Nat. Commun..

[41] Overholtzer M., Rao P.H., Favis R., Lu X.-Y., Elowitz M.B., Barany F., Ladanyi M., Gorlick R., Levine A.J. (2003). The presence of p53 mutations in human osteosarcomas correlates with high levels of genomic instability. Proc. Natl. Acad. Sci. USA.

[42] Mirabello L., Rao P.H., Favis R., Lu X.-Y., Elowitz M.B., Barany F., Ladanyi M., Gorlick R., Levine A.J. (2015). Germline TP53 variants and susceptibility to osteosarcoma. J. Natl. Cancer Inst..

[43] Hochane M., Trichet V., Pecqueur C., Avril P., Oliver L., Denis J., Brion R., Amiaud J., Pineau A., Naveilhan P. (2017). Low-Dose Pesticide Mixture Induces Senescence in Normal Mesenchymal Stem Cells (MSC) and Promotes Tumorigenic Phenotype in Premalignant MSC. Stem Cells.

[44] Wang L., Zhao Y., Liu Y., Akiyama K., Chen C., Qu C., Jin Y., Shi S. (2013). IFN-γ and TNF-α Synergistically Induce Mesenchymal Stem Cell Impairment and Tumorigenesis via NFκB Signaling. Stem Cells.

[45] Rhim J.S., Park D.K., Arnstein P., Huebner R.J., Weisburger E.K., Nelson-Rees W.A. (1975). Transformation of human cells in culture by *N*-methyl-*N*′-nitro-*N*-nitrosoguanidine. Nature.

[46] Pantic I. (2011). Cancer stem cell hypotheses: Impact on modern molecular physiology and pharmacology research. J. Biosci..

[47] Brennan M.Á., Renaud A., Amiaud J., Rojewski M.T., Schrezenmeier H., Heymann D., Trichet V., Layrolle P. (2014). Pre-clinical studies of bone regeneration with human bone marrow stromal cells and biphasic calcium phosphate. Stem Cell Res. Ther..

[48] Correa D., Somoza R.A., Lin P., Schiemann W.P., Caplan A.I. (2016). Mesenchymal stem cells regulate melanoma cancer cells extravasation to bone and liver at their perivascular niche: MSC/pericytes regulate bone and liver melanoma invasion. Int. J. Cancer.

[49] Caplan A.I. (2017). Mesenchymal Stem Cells: Time to Change the Name! Mesenchymal Stem Cells. Stem Cells Transl. Med..

[50] Xu W., Bian Z., Fan Q., Li G., Tang T. (2009). Human mesenchymal stem cells (hMSCs) target osteosarcoma and promote its growth and pulmonary metastasis. Cancer Lett..

[51] Spaeth E.L., Dembinski J.L., Sasser A.K., Watson K., Klopp A., Hall B., Andreeff M., Marini F. (2009). Mesenchymal Stem Cell Transition to Tumor-Associated Fibroblasts Contributes to Fibrovascular Network Expansion and Tumor Progression. PLoS ONE.

[52] Xing F., Saidou J., Watabe K. (2010). Cancer associated fibroblasts (CAFs) in tumor microenvironment. Front. Biosci. Landmark Ed..

[53] Blau O., Baldus C.D., Hofmann W.-K., Thiel G., Nolte F., Burmeister T., Türkmen S., Benlasfer O., Schümann E., Sindram A. (2011). Mesenchymal stromal cells of myelodysplastic syndrome and acute myeloid leukemia patients have distinct genetic abnormalities compared with leukemic blasts. Blood.

[54] Kurose K., Gilley K., Matsumoto S., Watson P.H., Zhou X.-P., Eng C. (2002). Frequent somatic mutations in PTEN and TP53 are mutually exclusive in the stroma of breast carcinomas. Nat. Genet..

[55] Kornicka K., Marycz K., Tomaszewski K.A., Marędziak M., Śmieszek A. (2015). The Effect of Age on Osteogenic and Adipogenic Differentiation Potential of Human Adipose Derived Stromal Stem Cells (hASCs) and the Impact of Stress Factors in the Course of the Differentiation Process. Oxid. Med. Cell. Longev..

[56] Gomez-Brouchet A., Illac C., Gilhodes J., Bouvier C., Aubert S., Guinebretiere J.-M., Marie B., Larousserie F., Entz-Werlé N., de Pinieux G. (2017). CD163-positive tumor-associated macrophages and CD8-positive cytotoxic lymphocytes are powerful diagnostic markers for the therapeutic stratification of osteosarcoma patients: An immunohistochemical analysis of the biopsies fromthe French OS2006 phase 3 trial. OncoImmunology.

[57] Abumaree M., al Jumah M., Pace R.A., Kalionis B. (2012). Immunosuppressive Properties of Mesenchymal Stem Cells. Stem Cell Rev. Rep..

[58] Studeny M., Marini F.C., Dembinski J.L., Zompetta C., Cabreira-Hansen M., Bekele B.N., Champlin R.E., Andreeff M. (2004). Mesenchymal Stem Cells: Potential Precursors for Tumor Stroma and Targeted-Delivery Vehicles for Anticancer Agents. JNCI J. Natl. Cancer Inst..

[59] Sun K., Gong C., Peng H., Fang H., Zhou J., Li J., Chen S., Zheng H. (2017). High CCL5 expression is associated with osteosarcoma metastasis and poor prognosis of patients with osteosarcoma. Mol. Med. Rep..

[60] Aggoune D., Sorel N., Bonnet M.-L., Goujon J.-M., Tarte K., Hérault O., Domenech J., Réa D., Legros L., Johnson-Ansa H. (2017). Bone marrow mesenchymal stromal cell (MSC) gene profiling in chronic myeloid leukemia (CML) patients at diagnosis and in deep molecular response induced by tyrosine kinase inhibitors (TKIs). Leuk. Res..

[61] Brennan M.Á., Renaud A., Gamblin A., D’Arros C., Nedellec S., Trichet V., Layrolle P. (2015). 3D cell culture and osteogenic differentiation of human bone marrow stromal cells plated onto jet-sprayed or electrospun micro-fiber scaffolds. Biomed. Mater..

