# Supplementary material for: Comparison of Tumor- and Bone Marrow-Derived Mesenchymal Stromal/Stem Cells from Patients with High-Grade Osteosarcoma
Source: Int J Mol Sci. 2018 Mar 1;19(3):707. doi: 10.3390/ijms19030707 (PMC5877568; doi:10.3390/ijms19030707)

SUPPLEMENTARY TABLES AND FIGURES

	patient	3		1		healthy	1	2					7		6		9		8	5		4	
	**primary cells**	**MSC**	**OSDC**	**OSDC**		**MSC**	**OSDC**	**OSDC**			**OSDC p5**	**MSC p5**	**MSC**	**OSDC**	**MSC**	**OSDC**	**OSDC p8**	**OSDC**	**OSDC**	**MSC**	**OSDC**	**MSC**	**OSDC**
	**culture selection**	**AD**	**SP**	**SP1**	**SP2**	**AD**		**SP1**	**SP2**	**SP3**	**AD**		**AD**		**AD**		**AD**	**AD**	**AD**	**AD**		**AD**	
CD34	hematopoietic progenitor antigen 1	1.0	1.0	**4.7**	**2.5**	0.8	**2.0**	1.0	1.4	1.3	1.0	1.0	1.0	1.0	1.0	1.0	1	1	1.4	2.6	1.8	1.0	1.5
CD45	tyrosine phosphase receptor	1.0	1.0	1.0	1.0	1.0	1.0	1.0	1.0	1.0	1.0	1.0	1.0	1.0	1.0	1.0	1	1	1.0	2.5	1.1	1.0	1.0
CD117	tyrosine kinase receptor	1.0	1.0	1.0	1.3	1.6	1.9	n.d.	n.d.	n.d.	n.d.	n.d.	n.d.	n.d.	n.d.	n.d.	n.d.	n.d.	n.d.	n.d.	n.d.	n.d.	n.d.
CD44	hyaluronic acid receptor	154.0	245.0	171.0	274.0	154.3	169.0	211	195	163	65	94	14.9	8.0	21.3	18.6	41.9	278	61.4	406	320.5	172	189.6
CD73	ecto-5' nucleotidase	66.0	104.0	66.0	103.0	65.7	127.8	n.d.	n.d.	n.d.	n.d.	n.d.	8.3	7.3	13.9	9.1	30.4	33.5	56.0	148	n.d.	76	n.d.
CD90	Thy1 Cell Surface Antigen	157.0	494.0	434.0	530.0	156.0	554.0	592	447	348	240	536	116.2	94.0	22.4	93.7	130	927	347.0	n.d.	53.6	n.d.	25.9
CD105	endoglin	112.0	47.0	45.0	35.0	11.6	83.0	94	50	33	55	79	10.9	26.9	140.7	72	38.8	182	55.3	198	104	73.7	112.3

Supplementary Table 1: Ratio of mean fluorescent intensity (MFI) for cluster of differentiation (CD) antigens. Primary cells were derived from bone marrow (MSC) or OS samples (OSDC) and obtained from sphere (SP) formed in non-adherence culture condition or from cell amplification in adherence (AD) culture condition. Passage number (p5, p8) is indicated when cells were analysed later than passage ¾. Ratios of MFI are indicated and correspond to MFI of cells with CD-directed antibody / MFI of cells incubated with isotype antibody. Tested CD are indicated with corresponding protein name. Not determined (n.d.). The color change of the columns indicates that flow cytometry was performed at different time.

Supplementary Figure 1 : representative images of optical microscopic observation of OSDC-2 culture in anchorage-independent condition without (a) or with bFGF (b) . Spheres were obtained only with bFGF. A representative sphere is shown (c).

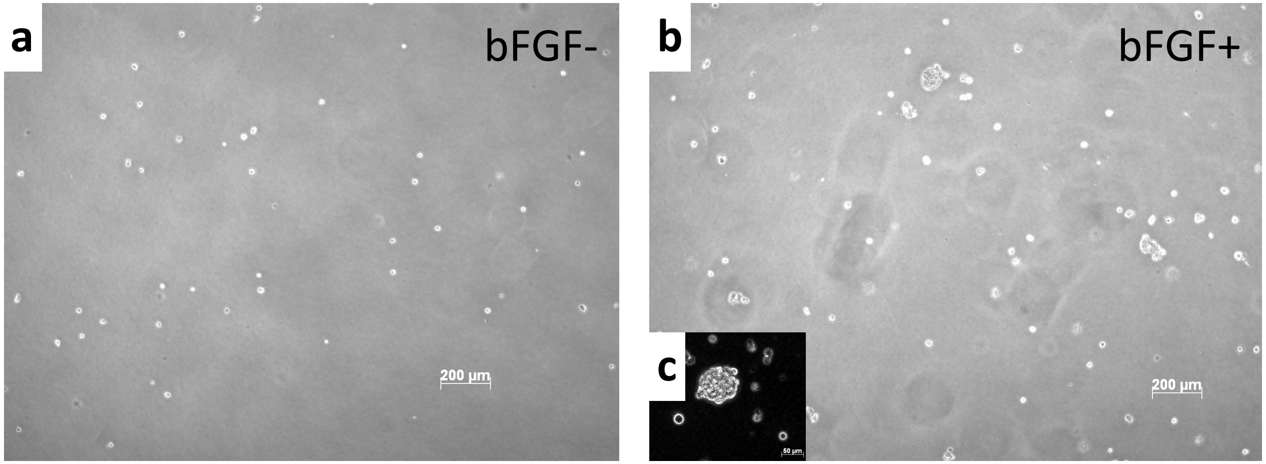

Supplementary Figure 2 : normal karyotype for MSC-3, OSDC-1 and OSDC-8. For comparison, a representative osteosarcoma karyotype is shown below (Gillette JM, Gibbs CP and Nielsen-Preiss SM. Establishment and characterization of OS 99-1. a cell line derived from a highly aggressive primary human osteosarcoma. In Vitro Cell Dev Biol Anim. 2008 Mar-Apr;44(3-4):87-95.)

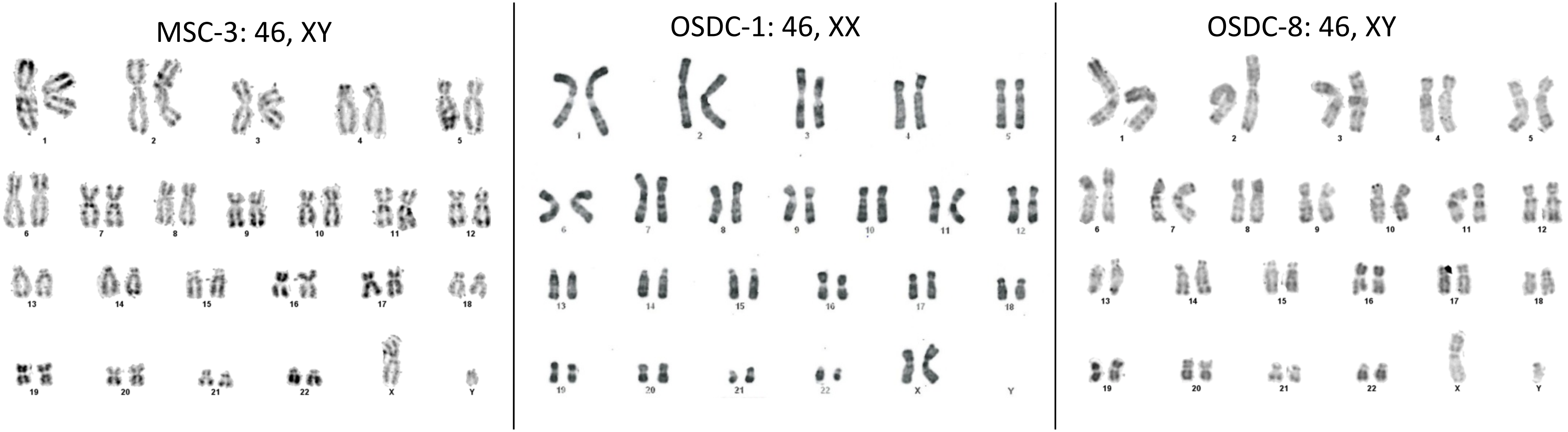

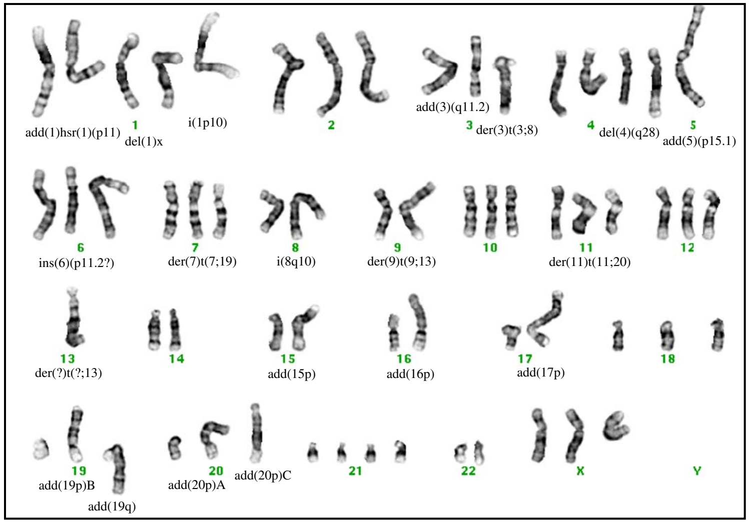

Supplementary Figure 3 : Co-injection of OS-inducing cells (MNNG-HOS cells (HOS)) with osteosarcoma derived cells (OSDC) in athymic mouse. Individual tumor volumes are represented as dots and mean tumor volumes by a line. ns: p>0.05, *: 0.05>p>0.0, **: 0.01>p>0.001.

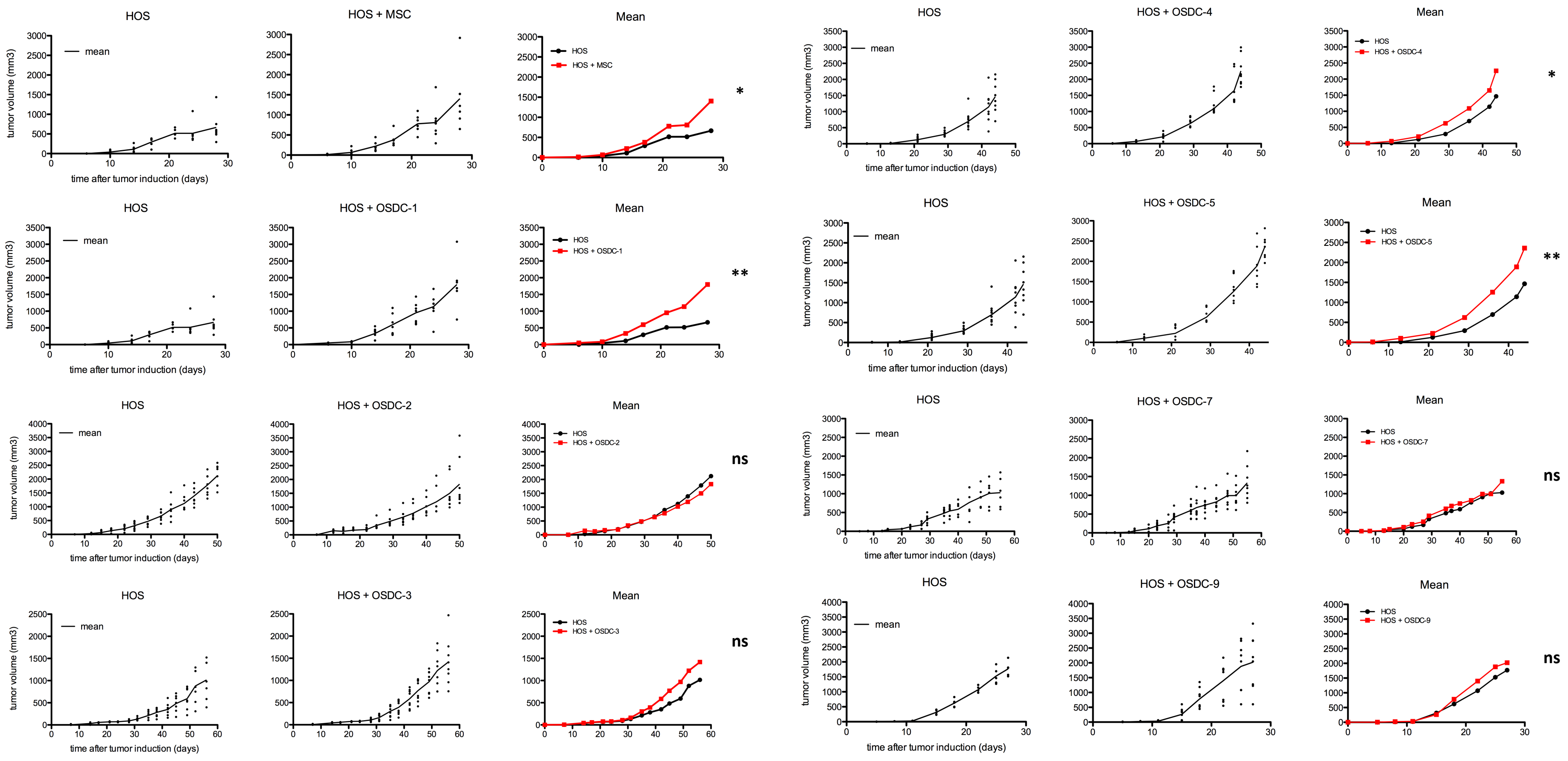

Supplementary Figure 4 : Quantification of different cytokines or growth factors using multiplex immunoassay in medium conditioned by healthy donor bone marrow MSC (MSC) and OSDC-1. Interleukins (IL), chemokine ligands (CCL, RANTES (CCL5) and CXCL), growth factors (fibroblast GF-2, vascular endothelial GF-A, platelet-derived GF-bb, tumor necrosis factor alpha, interferon gamma, leukemia inhibitory factor) and adipokines (leptin, resistin, visfatin) were measured. #: not detected.

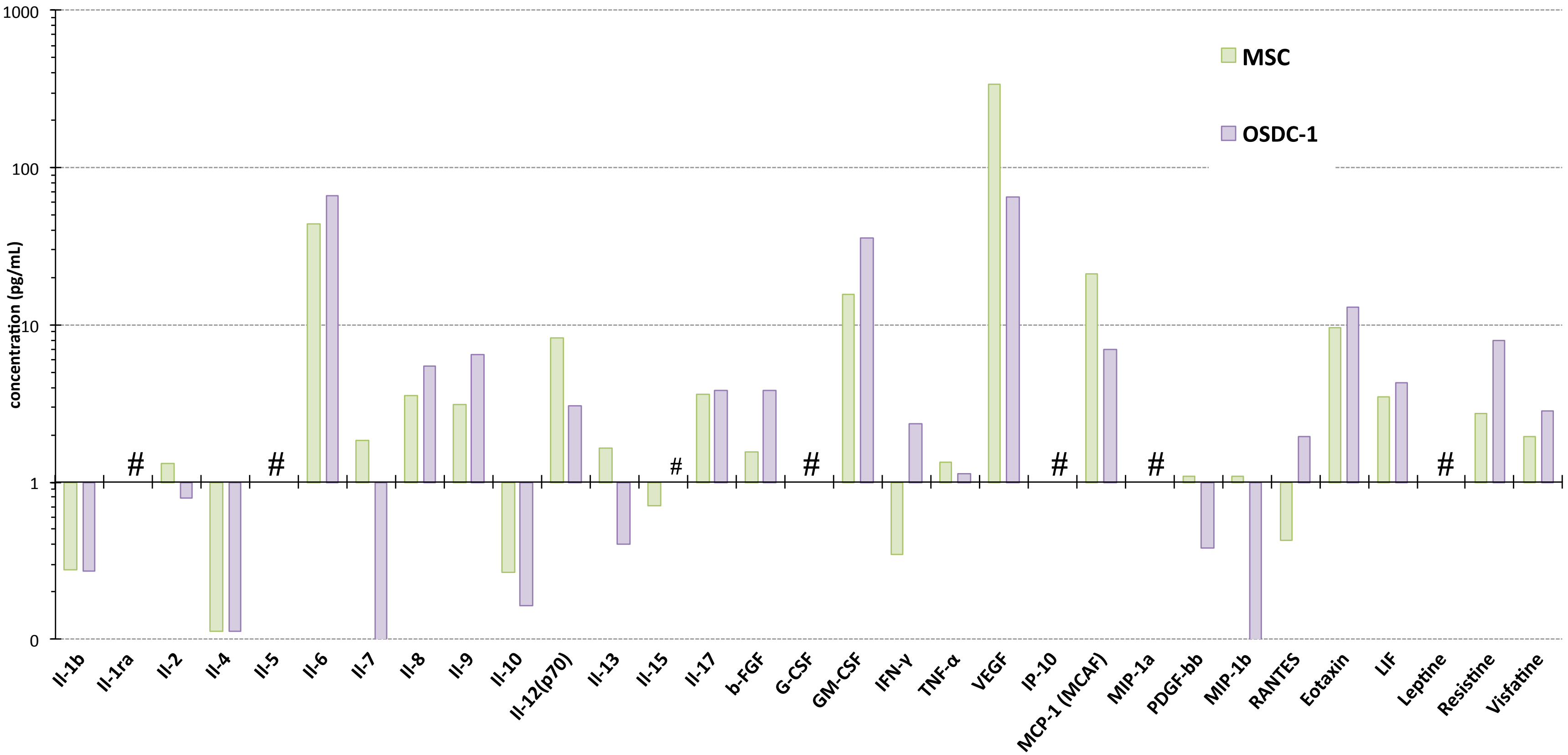

Supplement: Supplementary file 1 [file ijms-19-00707-s001.pdf]
